# Biomarkers in Heart Failure and Associated Diseases

**DOI:** 10.1155/2019/8768624

**Published:** 2019-02-07

**Authors:** Andrea Salzano, Alberto M. Marra, Marco Proietti, Valeria Raparelli, Liam M. Heaney

**Affiliations:** ^1^Department of Cardiovascular Sciences and NIHR Leicester Biomedical Research Centre, University of Leicester, Glenfield Hospital, Groby Road, LE3 9QP Leicester, UK; ^2^Department of Translational Medical Sciences, Federico II University, Via Pansini 5, Naples 80138, Italy; ^3^IRCCS SDN, Naples, Italy; ^4^Istituto di Ricerche Farmacologiche Mario Negri IRCCS, Via Mario Negri 2, 20156 Milan, Italy; ^5^Department of Internal Medicine and Medical Specialties, Sapienza University of Rome, Viale del Policlinico 155, 00161 Rome, Italy; ^6^McGill University Health Centre Research Institute, Centre for Outcomes Research and Evaluation, 5252 De Maisonneuve, Montreal, QC, Canada H4A 3S9; ^7^Department of Experimental Medicine, Sapienza University of Rome, Viale del Policlinico 155, 00161 Rome, Italy; ^8^School of Sport, Exercise & Health Sciences, Loughborough University, Loughborough LE11 3TU, UK

Despite considerable improvement in the management of heart failure (HF), unsustainable levels of morbidity and mortality coupled with an increasing economic and social burden have been observed over the previous three decades [1]. A rational explanation of this is the fact that no single pathophysiologic paradigm of HF has been clarified, resulting in failure of our current models to completely explain disease progression [2].

Classically, seven categories of biomarkers in HF have been described, reflecting the different pathophysiological pathways involved in disease progression [3]. These include myocardial stretch, myocyte injury, matrix remodelling, inflammation, neurohumoral activation, oxidative stress, and indices of renal dysfunction [4]. Moreover, growing evidence supports the key role of alternative pathophysiological pathways (e.g., the gastrointestinal system, the anabolic/catabolic imbalance, and multiple hormonal deficiency syndrome), with ever-increasing identifications of novel biomarkers that demonstrate their importance in HF [5–9].

In this context, a growing interest in multimarker approaches to biomarker panels to assess multiple pathophysiologic pathways has been realised, including the combined use of proteins, lipids, metabolites, hormones, and genetic markers [10].

Owing to these recent advances in biomarker research, the aim of this special issue was to focus on the role of biomarkers in HF and associated diseases.

Ischemic heart disease is to date the most frequent cause of HF [2], with atherosclerosis the major pathophysiological mechanism. In this issue, L.-D. Mocan Hognogi et al. reviewed the role of adipokines (in particular visfatin, apelin, leptin, and resistin) as biomarkers of ischemic cardiac disease and concluded that “*there is no doubt that inflammation is viewed as an important pathophysiological step in the development of atherosclerosis*.”

Importantly, the identification of patients at high risk of poor prognosis is one of the principal aims of current clinical research [11]. With this regard, M. Alavi-Moghaddam et al. conducted a pilot study involving 21 patients diagnosed with acute myocardial infarction and demonstrated that plasma levels of microRNA-208b, of which levels of expression have been demonstrated to be increased in the blood of patients with acute myocardial infarction, were 2-fold higher in patients who died after 6 months than in those which survived.

Further, J. Banach et al. investigated plasma concentration of procalcitonin (PCT) in 130 patients with chronic HF with reduced ejection fraction, assessing its prognostic value during a 24-month follow-up period. Indeed, PCT levels were significantly higher in HF patients when compared to a control group. Further, Kaplan-Meier survival curves revealed that patients with PCT in the highest quartile had a significantly reduced probability of survival. This is additional evidence supporting the role of inflammation in HF [7].

Diabetic cardiomyopathy (DCM) is a common cardiac dysfunction, affecting approximately 12% of diabetic patients, and is featured by ventricular diastolic and (or) systolic dysfunction. N. Li et al. provided a comprehensive and novel illustration of gene expression profiles to identify differentially expressed genes in myocardial tissue, which may play critical roles in the occurrence and development in patients with DCM. This is of great interest considering that diabetes mellitus has been described in approximately 20-25% of HF patients [5, 12].

HF is a progressive condition in which myocardial damage, caused by cardiovascular risk factors, leads to the development of myocardial dysfunction. Thus, an ever-worsening condition is present until the patient eventually develops end-stage heart failure. Heart transplantation is the only survival option for end-stage patients [2]. Cardiac allograft vasculopathy (CAV) is the leading cause of cardiovascular adverse events during follow-up of heart transplantation. S. Mirabet et al. demonstrated that high-sensitivity cardiac troponin T, measured during a long-term follow-up, appears as a helpful biomarker to identify patients at low risk of adverse CV outcomes. On the other hand, the soluble form of AXL (sAXL) and a biomarker of endothelial dysfunction was not able to predict outcome.

In conclusion, this issue collected novel findings and shed light upon the role of biomarkers in HF.
